# Inhibition of urease activity by different compounds provides insight into the modulation and association of bacterial nickel import and ureolysis

**DOI:** 10.1038/s41598-020-65107-9

**Published:** 2020-05-22

**Authors:** Simon Svane, Jens Jakob Sigurdarson, Friedrich Finkenwirth, Thomas Eitinger, Henrik Karring

**Affiliations:** 1 Department of Chemical Engineering, Biotechnology and Environmental Technology, University of Southern Denmark, Campusvej 55, 5230 Odense M, Denmark; 2 Institut für Biologie/Mikrobiologie, Humboldt-Universität zu Berlin, Unter den Linden 6, 10099 Berlin, Germany

**Keywords:** Biochemistry, Biotechnology, Microbiology

## Abstract

The nickel-dependent urease enzyme is responsible for the hydrolysis of urea to ammonia and carbon dioxide. A number of bacteria produce urease (ureolytic bacteria) and are associated with various infectious diseases and ammonia emissions from agriculture. We report the first comprehensive comparison of the inhibition of urease activity by compounds analysed under the same conditions. Thus, 71 commercially available compounds were screened for their anti-ureolytic properties against both the ureolytic bacterium *Klebsiella pneumoniae* and purified jack bean urease. Of the tested compounds, 30 showed more than 25% inhibition of the ureolytic activity of *Klebsiella pneumoniae* or jack bean urease, and among these, carbon disulfide, N-phenylmaleimide, diethylenetriaminepentaacetic acid, sodium pyrrolidinedithiocarbamate, 1,2,4-butanetricarboxylic acid, tannic acid, and gallic acid have not previously been reported to possess anti-ureolytic properties. The diverse effects of metal ion chelators on ureolysis were investigated using a cellular nickel uptake assay. Ethylenediaminetetraacetic acid (EDTA) and dimethylglyoxime (DMG) clearly reduced the nickel import and ureolytic activity of cells, oxalic acid stimulated nickel import but reduced the ureolytic activity of cells, 1,2,4-butanetricarboxylic acid strongly stimulated nickel import and slightly increased the ureolytic activity of cells, while L-cysteine had no effect on nickel import but efficiently reduced the ureolytic activity of cells.

## Introduction

**Figure 1:**
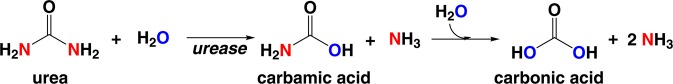
Overall urea hydrolysis reaction catalysed by urease. Urease catalyses the hydrolysis of urea to carbamic acid and NH_3_. Carbamic acid spontaneously decomposes in solution to yield carbonic acid and an additional molecule of NH_3_. Nitrogen atoms (N) are shown in red and oxygen atoms from water (O) are shown in blue.

Urease (EC 3.5.1.5), a dinickel enzyme, catalyses the hydrolysis of urea to carbonic acid (H_2_CO_3_) and ammonia (NH_3_) via the formation of carbamic acid (H_2_NCOOH) (Fig. 1)^1^. In aqueous solutions, the carbonic acid and NH_3_ are in equilibrium with bicarbonate (HCO_3_^−^) and ammonium (NH_4_^+^) ions, respectively. Urease is produced by bacteria, fungi, plants, and invertebrates, and its primary structure and active site are surprisingly conserved among different species^2^. The active site of urease contains two Ni^2+^ ions, which are bridged by a hydroxyl group and a carbamylated lysine.

The consequences of urease-driven urea hydrolysis and the accompanying pH increase caused by NH_3_ production are widespread and, therefore, are relevant in several aspects. The human pathogenic bacterium *Helicobacter pylori* (*H. pylori*), which colonizes the stomach and is linked to diseases such as gastric ulcers, gastritis and stomach cancer^3,4^, produces large amounts of urease and degrades urea to survive the acidic gastric environment^5^. In the oral cavity, *Streptococcus salivarius* produces ammonia from urea hydrolysis in response to low pH, leading to dental plaque and calculus deposition^6^. Other ureolytic bacteria, such as *Klebsiella pneumoniae* (*K. pneumoniae*) and *Proteus mirabilis*, are involved in pneumonia, kidney stone formation, and urinary tract infections^7,8^. Urease activity is an important pathogenic factor, and ten out of twelve antibiotic-resistant pathogens designated “priority pathogens” by the WHO^9^ are, in fact, ureolytic. In agriculture, ureolytic bacteria present in animal faeces and soil are responsible for significant losses of nitrogen from manure slurry and synthetic urea fertilizers, respectively. Nitrogen is lost as harmful emissions of ammonia to the environment, increasing the formation of hazardous atmospheric PM_2.5_ particles^10–12^. The many challenges associated with ureolytic bacteria fuel continuous research into strategies for controlling the ureolytic activity of microorganisms. Inhibition of urease is often part of the medical treatment of infections by ureolytic bacteria^13^. This inhibition is normally carried out by small molecular inhibitors that are safe and metabolically stable *in viv*o. In agriculture, other small-molecule urease inhibitors, predominantly N-(n-butyl)thiophosphoric triamide (NBPT), are used as additives in synthetic urea fertilizer formulations to reduce the loss of fertilizer nitrogen from fields by slowing down the conversion of stable urea to volatile NH_3_^14^.

These different application areas of urease inhibitors indicate a need for a diverse “panel of compounds” as pharmaceutical inhibitors must have other properties than the urease inhibitors used in agriculture. Most commercially available anti-ureolytic compounds can be placed in one of three main categories according to their mode of action. Some inhibitors act by direct binding to Ni^2+^ in the active site of urease, e.g., phosphodiamidates, fluoride, and hydroxyurea. The second category includes compounds such as quinones that covalently modify a specific cysteine present in the mobile “flap” covering the dinickel centre of the enzyme, which consequently locks the flap in a specific conformation leading to the loss of catalytic activity. The third category of compounds consists of metal ion chelators, such as EDTA, which can sequester Ni^2+^, inhibiting the formation of the dinickel centre and thereby active urease.

Many urease inhibitors of varying efficiency, cost, toxicity, and stability are known, ranging from elemental ions (e.g., Hg^2+^ and Ag^+^)^15,16^ and inorganic ions (e.g., boric acid)^17^ to organic compounds (e.g., acetohydroxamic acid and hydroquinone)^18,19^. Additionally, a number of readily available organic compounds share structural commonalities with known urease inhibitors, but no studies have been published concerning their anti-ureolytic activity. These compounds may not have been tested against urease or ureolytic organisms, or they may have simply not been reported due to a lack of activity. In many cases, only a few structurally related compounds have been investigated. Previous studies have analysed compounds under different experimental conditions, making it difficult to compare the anti-ureolytic activity of the compounds tested. In this comparative study, a recently developed assay^20^ was used to test the ability of 71 carefully selected compounds to inhibit the ureolytic activity of *K. pneumoniae* and purified jack bean urease (JBU) under the same conditions. Both potential and known inhibitors were evaluated based on their ability to reduce the rate of pH increase and the overall pH increase in urea solutions. Compounds showing little or no anti-ureolytic effect are reported in the supporting information to aid in the design of future studies of urease inhibitors.

## Results and Discussion

Eighty-four compounds (Table 1) were chosen for evaluation of their anti-ureolytic effect using both a bacterial and an enzyme activity assay described previously^20^. The 84 compounds were carefully selected on the basis of previously being reported to have anti-ureolytic properties or because they share structural similarities with known urease inhibitors (Table 1). A number of metal ion-chelating compounds were tested for their ability to reduce bacterial ureolysis by sequestering Ni^2+^. During the selection of screening candidates, preference was given to stable and commercially available compounds and preferably compounds of low human -, animal -, and environmental toxicity. Especially inorganic toxic substances such as mercury were excluded, while a few organic chemicals such as pyrocatechol and p-benzoquinone were included in the screening despite their toxicity.

**Table 1:** List of the 84 compounds selected for comparison and evaluation of their anti-ureolytic activity.

Compound name			
1,2,4-butanetricarboxylic acid	creatinine	L-cysteine methyl ester ∙ HCl	potassium permanganate
1-(3-carboxyphenyl)-2-thiourea	cysteamine ∙ HCl	L-glutamine	pyrocatechol
1,4-dithiothreitol, *DTT*	diethylenetriaminepentaacetic acid (in 40% EtOH)	L-histidine	rhodanine-3-acetic acid^b^
1,4,8,11-tetraazacyclotetradecane, *cyclam*^a^	dimethylformamide, *DMF*	magnesium sulfate	silver nitrate
2-bromo-2-nitropropane-1,3-diol, *Bronopol*	dimethylglyoxime, *DMG* (in 99% EtOH)	methylurea	sodium dihydrogenphosphate ∙ 2H_2_O
2-mercaptoethanol	ethacrynic acid (in 99% EtOH)	murexide^c^	sodium fluoride
2-thiobarbituric acid (in 20% EtOH)	ethylenediaminetetraacetic acid, *EDTA*	N-(2-acetamido)iminodiacetic acid	sodium hypophosphite ∙ 6H_2_O
2,2’-thenoin	etidronic acid ∙ H_2_O	N-(2-hydroxyethyl)ethylenediamine^a^	sodium pyrrolidinedithiocarbamate
2,5-dichloro-1,4-benzoquinone^b^	ferric dimethyldithiocarbamate^b^	N-(4-hydroxyphenyl)glycine (in 99% EtOH)	sodium sulfite
2,5-dimethyl-1,4-benzoquinone (in 99% EtOH)	formamide	N-(n-butyl)thiophosphoric triamide, *NBPT*	sodium tetraborate
3,3’-methylene-bis-(4-hydroxycoumarin)	furoin^b^	N,N-diphenylurea^b^	sodium tetrathionate ∙ 2H_2_O
4-bromophenylboronic acid (in 50% EtOH)	gallic acid ∙ H_2_O (in 33.3% EtOH)	N,N’-dimethylurea	sodium thiocyanate
acetylthiourea	guanidine hydrochloride	nitrilotriacetic acid, *NTA*	sodium thiosulfate
acetohydroxamic acid, *AHA*	hexaminecobalt(III) chloride	N-phenylmaleimide (in 99% EtOH)	sulfamic acid
allantoin	hydroquinone	oxalic acid	tannic acid
alloxan	hydroxyurea	*p*-benzoquinone	triethylenetetraamine
allylthiourea	Imidazole	*p*-chlorophenol (in 96% EtOH)	tetrachloro-1,4-benzoquinone^b^
bismuth(III) gallate basic hydrate^b^	iminodiacetic acid, *IDA*	phenyl dichlorophosphate	tetramethylthiuram disulfide^b^
boric acid	L-arginine	phenyl phosphorodiamidate, *PPDA*	tetramethylthiuram sulfide^b^
carbon disulfide	L-cysteine	potassium disulfite	thiourea
creatine ∙ H_2_O	L-cysteine ethyl ester ∙ HCl	potassium hexacyanoferrate(III)	uric acid^b^

Before being tested in the urease activity assays, compounds were dissolved in pure water unless otherwise stated in parentheses. For compounds that are commonly abbreviated or have trade names, the abbreviation/name is noted in italics. The 13 compounds marked with a superscript letter were discarded from further analyses due to incompatibility with the ureolytic activity assays.

^a^Compound was sufficiently alkaline to cause an interfering increase in the absorbance at 557 nm.

^b^Compound was insufficiently soluble in water, water:EtOH mixtures, and 99% EtOH.

^c^Solutions of the compound were coloured and absorbed strongly at 557 nm.

### Measuring and comparing the anti-ureolytic effects of selected compounds

**Figure 2:**
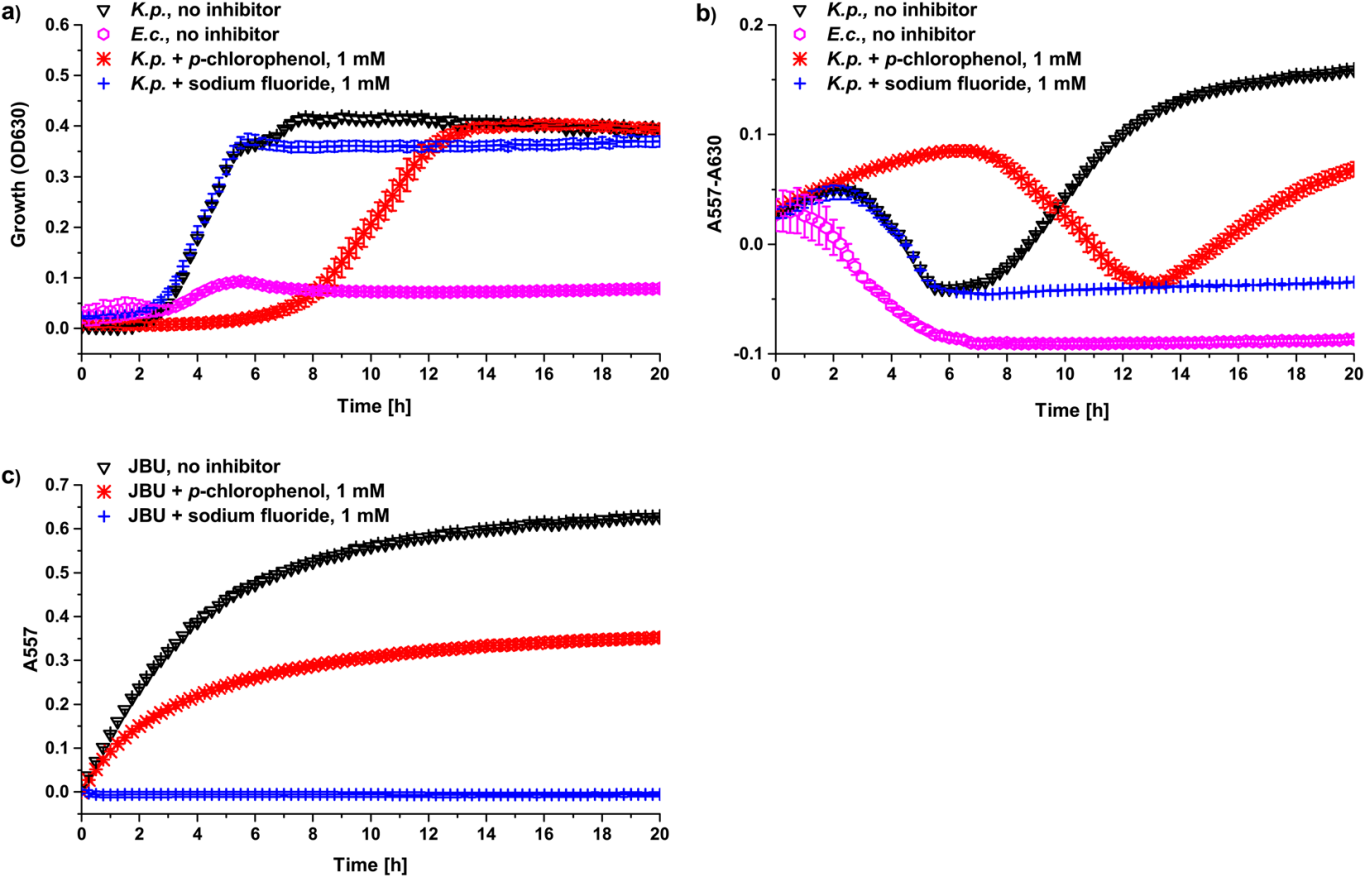
Typical growth and pH-related absorbance curves from ureolytic activity assays in the absence and presence of urease inhibitors. The growth (**a**) and ureolytic activity (**b**) of cultured *K. pneumoniae* (*K.p*.) were monitored by measuring the optical density (OD630) and pH increase (A557 – A630), respectively. *E. coli* K12 MG1655 (*E.c*.) was included as a non-ureolytic control. The enzyme activity of jack bean urease (JBU) was followed by the pH increase (A557) of the solution (**c**). Both bacterial and enzyme assays were applied to test the ability of each compound to inhibit urease activity in M9U medium containing 40 mM urea. Growth and ureolytic activity curves are shown for four different conditions: non-ureolytic *E.c*. control (), *K.p*. or JBU without inhibitor (), *K.p*. or JBU with 1 mM *p*-chlorophenol (), and *K.p*. or JBU with 1 mM sodium fluoride (). Data are shown as the mean ± SD (n = 3).

The ability of each compound to influence ureolytic activity was determined by monitoring the pH increase caused by ammonia production in solutions containing *K. pneumoniae* or JBU with 40 mM urea (Fig. 2) as previously described^20^. Briefly, the increase in absorbance of a pH indicator at 557 nm (A557) was used as a measure of the pH increase caused by ureolysis^20–22^. To compare the anti-ureolytic ability of each compound against *K. pneumoniae*, the onset of pH increase, rate of pH increase, and the final corrected absorbance at 557 nm (Final A557 – A630) were recorded and compared to a reference where no inhibitor was added (Supplementary Table S1 and Table 2). The end of the lag phase (onset of growth), rate of growth, and final OD630 were also recorded to determine what influence, if any, the various inhibitors had on bacterial growth (Supplementary Table S1). In the enzyme activity assay, the initial rate of pH increase and the final pH-related absorption (Final A557) of each inhibitor were also recorded and compared with those of a reference containing JBU without inhibitor present (Fig. 2c, Supplementary Table S2, and Table 2).

**Table 2:** Anti-ureolytic effects of selected compounds screened against *K. pneumoniae* and jack bean urease.

Category of anti-ureolytic mechanism	Anti-ureolytic compound	Concentration of compound [mM]	*K. pneumoniae*		Jack bean urease	
			Change in rate of pH (A557-A630) increase	Change in final pH (Final A557-A630)	Change in initial rate of pH (A557) increase	Change in final pH (Final A557)
Substrate analogue	4-bromophenylboronic acid	0.02	N/A^a^	N/A^a^	−20.0 ± 8.8%	−51.7 ± 13.5%
	acetohydroxamic acid	0.1	−49.9 ± 10.4%	−8.8 ± 16.2%	−62.2 ± 4.1%	−70.1 ± 1.7%
	boric acid	0.1	−7.5 ± 2.1%	4.3 ± 2.5%	−36.7 ± 4.3%	−48.7 ± 5.7%
	hydroxyurea	0.1	−9.6 ± 1.2%	<1 ± 4.0%	−38.0 ± 4.4%	−63.6 ± 1.3%
Transition state analogue	NBPT	0.02	−100%	−100%	−39.3 ± 4.6%	−59.8 ± 3.3%
	PPDA	0.007	−100%	−100%	−100%	−100%
Binding to Ni in active site	carbon disulfide	0.03	−3.3 ± 2.9%	−2.8 ± 4.2%	−42.6 ± 12.1%	−27.5 ± 2.4%
	sodium fluoride	1.0	−100%	−100%	−100%	−100%
Binding of Ni in active site/disulfide formation	2-mercaptoethanol	1.0	−100%	−100%	−5.8 ± 12.7%	<1.0 ± 2.9%
	cysteamine hydrochloride	0.1	−4.2 ± 4.8%	<−1 ± 2.9%	40.6 ± 10.9%	−36.4 ± 1.1%
Oxidation of “flap” cysteine	2-bromo-2-nitropropane-1,3-diol	0.1	−100%	−100%	−63.4 ± 8.6%	−88.8 ± 2.4%
	potassium permanganate	0.1	5.5 ± 3.7%	−64.3 ± 3.0%	−90.3 ± 2.2%	−92.2 ± 1.9%
Michael acceptor covalently modifying “flap” cysteine	2,5-dimethyl-1,4-benzoquinone (in 99% EtOH)	0.01	−2.3 ± 3.2%	−3.8 ± 4.2%	−81.4 ± 3.7%	−81.6 ± 1.4%
	*p*-chlorophenol (in 99% EtOH)	0.1	−4.1 ± 3.0%	−6.6 ± 4.6%	−15.6 ± 8.2%	−38.9 ± 3.8%
	ethacrynic acid (in 99% EtOH)	0.1	−5.3 ± 6.9%	−9.3 ± 4.6%	−75.1 ± 6.1%	−28.1 ± 14.9%
	hydroquinone	0.1	7.8 ± 15.6%	1.0 ± 8.6%	−90.9 ± 1.8%	−94.5 ± 0.3%
	N-(4-hydroxyphenyl)glycine	0.001	9.9 ± 2.4%	−21.8 ± 3.5%	−9.5%±10.3%	−45.0%±6.5%
	N-phenylmaleimide (in 99% EtOH)	0.04	1.2 ± 6.1%	−11.2 ± 4.3%	−100%	−100%
	p-benzoquinone	0.02	1.7 ± 3.4%	−1.7 ± 4.7%	−100%	−100%
	pyrocatechol	0.1	9.1 ± 1.5%	2.2 ± 1.1%	−100%	−100%
“Flap” cysteine modification	silver nitrate	0.1	−100%	−100%	−100%	−100%
Chelator	1,2,4-butanetricarboxylic acid	0.1	1.2 ± 1.0%	−2.7 ± 2.0%	−29.8 ± 11.8%	−25.4 ± 10.4%
	diethylenetriaminepentaacetic acid	0.04	−100%	−100%	218.1 ± 49.3%	187.8 ± 31.0%
	EDTA	0.07	−100%	−100%	285.2%±100.2%	155.3%±28.1%
Multi-action, e.g., precipitation of proteins	tannic acid	0.1	−1.4 ± 0.7%	−18.4 ± 1.3%	−100%	−100%
Unknown	gallic acid	0.06	9.3 ± 2.4%	−18.8 ± 3.4%	−23.5 ± 10.0%	−64.0 ± 2.8%
	L-cysteine	1.0	−100%	−100%	254.6%±34.2%	162.4%±27.8%
	L-cysteine methyl ester hydrochloride	1.0	−100%	−100%	9.2%±6.2%	14.5%±13.0%
	L-cysteine ethyl ester hydrochloride	1.0	−100%	−100%	17.4 ± 6.1%	30.3 ± 15.9%
	sodium pyrrolidinedithiocarbamate	0.1	N/A^a^	N/A^a^	39.1 ± 9.4%	−25.8 ± 1.7%

The 30 most effective compounds are categorized according to their proposed anti-ureolytic mechanism. The listed concentration for each compound is the lowest tested concentration giving a significant anti-ureolytic effect (>25% reduction in the rate of pH increase and/or the final pH increase) in 40 mM urea solutions. The effects of the compounds on the rate of pH increase and final pH increase (Final A557 – A630) relative to the non-inhibited negative control are reported. Values are given as the ratio of the means ± SEM (n = 3). SEM was estimated using the Delta method. Full inhibition (Final A557 – A630 ≤ 0) is listed as a change of −100% relative to the final absorbance of the negative control. N/A = not available.

^a^4-Bromophenylboronic acid and sodium pyrrolidinedithiocarbamate showed a significant pH increase in the bacterial assay. However, both compounds showed antibacterial activity, and no growth was observed. Therefore, it is unlikely that the observed increase in absorbance was related to ureolysis. This effect was not observed in experiments with JBU.

Of the 84 compounds first selected to be tested in the ureolytic activity assays, 13 were discarded due to compatibility issues with the assay (Table 1). Thus, two compounds were too alkaline and significantly changed the phenol red absorption at 557 nm immediately after addition; ten compounds were insoluble in water, water/ethanol mixes or pure ethanol, and one compound gave rise to a strongly coloured solution that absorbed light in the same region as phenol red. The remaining 71 compounds were screened against *K. pneumoniae* and JBU, and their anti-ureolytic effects were evaluated based on their ability to delay the onset, reduce the rate, and lower the final value of the pH increase (Supplementary Table S1 and Supplementary Table S2). The assessment revealed that 30 compounds display significant anti-ureolytic effects (defined here as>25% reduction in the rate of pH increase and/or the final pH increase) on either *K. pneumoniae* and/or JBU (Table 2). In addition to well-known urease inhibitors, some unknown or less characterized anti-ureolytic agents were found among the effective inhibitors of urease activity (Table 2).

Some of the effective anti-ureolytic compounds were very selective and only reduced the ureolytic activity of either *K. pneumoniae* or the pure urease enzyme. Compounds that cannot cross the bacterial membrane or that are effectively catabolized or highly reactive typically had less of an effect against bacteria than the free enzyme. Other compounds, such as diethylenetriaminepentaacetic acid, EDTA, and L-cysteine, significantly increased the activity of pure urease but reduced the ureolytic activity of *K. pneumoniae* (Table 2). The screening revealed some unique observations for compounds of the different categories of anti-ureolytic mechanisms.

### Substrate analogue urease inhibitors

**Figure 3:**
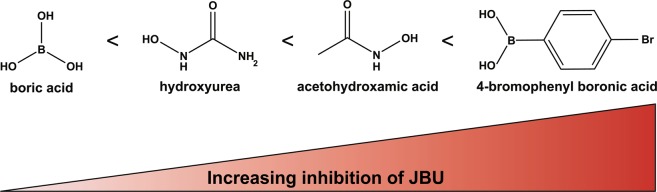
Structures of the most efficient substrate analogue urease inhibitors. The substrate analogues are ordered according to increasing inhibition of pure JBU at 0.1 mM concentration.

Urease inhibitors categorized as “substrate analogues” share structural similarity with the substrate urea and as such are usually competitive reversible inhibitors of urease^23^. One consequence of this is a lack of inhibitory efficiency in environments containing high amounts of urea, including animal manure slurry. Among the 13 screened substrate analogues, four showed anti-ureolytic ability at the concentrations applied here (Supplementary Table S1, Supplementary Table S2, and Table 2). A common trait among the substrate analogues was low inhibition of *K. pneumoniae* ureolytic activity but significant inhibition of pure urease (Fig. 3). An explanation could be that the urea analogues have trouble crossing the bacterial membrane and/or are metabolized rapidly by the bacteria.

The compound 4-bromophenyl boronic acid has been reported previously as a good inhibitor of urease^24,25^, and this observation was confirmed in the present study. The ureolytic activity of JBU was reduced by 51.7 ± 13.5% at an inhibitor concentration of 20 µM in 40 mM urea (Table 2, Fig. 3). To the best of our knowledge, the effect of 4-bromophenyl boronic acid on ureolytic bacteria has not been reported previously. Our results showed 100% growth inhibition of *K. pneumoniae* (OD630) when only 20 µM inhibitor was present in the medium, making 4-bromophenyl boronic acid a potentially useful antibacterial agent (Supplementary Table S1). Acetohydroxamic acid decreased the pH change of JBU by 70.1 ± 1.7% at 0.1 mM but did not affect the overall pH change of *K. pneumoniae*. Acetohydroxamic acid was, however, able to slow down the rate of ureolysis by *K. pneumoniae*, indicating that it is able to cross the membrane, but acetohydroxamic acid only effectively inhibited intracellular urease at relatively higher concentrations. Boric acid and hydroxyurea are both well-known urease inhibitors^18,26^, and while both compounds inhibited JBU, they failed to influence *K. pneumoniae* except at high concentrations (Supplementary Table S1, Supplementary Table S2, and Table 2).

### Transition-state Analogues PPDA and NBPT

Both N-(n-butyl)thiophosphoric triamide (NBPT) and phenyl phosphorodiamidate (PPDA) are well-characterized transition-state analogues of urea and are generally considered to be among the most efficient inhibitors of urease^27,28^. Both compounds have relatively low solubility in water, but they still inhibited ureolysis of *K. pneumoniae* by 100% and only slightly reduced the growth (Supplementary Table S1 and Table 2). Interestingly, only PPDA was 100% effective against free enzyme (JBU), even though the concentration of NBPT was nearly 3 times higher than the concentration of PPDA (Table 2). Thus, NBPT only reduced the rate of pH increase and the final pH change by 39.3 ± 4.6% and 59.8 ± 3.3%, respectively (Table 2). This is most likely because the oxidized form of NBPT (NBPTO) has been shown to be a much stronger urease inhibitor than NBPT^27,29^, whereas PPDA is an active inhibitor.

### Nickel Ligand Binding in the Active Site of Urease

Carbon disulfide (CS_2_) has not previously been investigated in relation to urease inhibition but was included in the assay because it is known to form relatively stable complexes with a variety of transition metals^30^. CS_2_ did not significantly inhibit *K. pneumoniae* ureolysis at any of the screened concentrations, but it did reduce the activity of JBU. Thus, in the reaction with JBU, at ∼30 µM CS_2_, the rate of pH increase was lowered by 42.6 ± 12.1%, and the final pH was reduced by 27.5 ± 2.4% (Table 2). Surprisingly, only the lowest concentration (28.5 µM) showed this effect, suggesting that competing reactions take place at higher concentrations of carbon disulfide (Supplementary Table S2). Sodium fluoride (NaF) is a pseudo-uncompetitive inhibitor^31^ of urease, and it showed good inhibition of both *K. pneumoniae* and JBU at a 1 mM concentration with a 100% decrease in the final pH (Table 2). At lower concentrations of NaF, only JBU was partially inhibited (app. 30% reduction in Final A557), while the ureolytic activity of bacteria remained unaffected (Supplementary Table S1 and Supplementary Table S2).

### Active Site Ni-binding and Disulfide Formation

Thiol compounds can potentially inhibit urease by both binding to the active site Ni and forming mixed disulfides with the catalytically important cysteine residue (Cysα319 in *K. pneumoniae* urease and Cys592 in JBU)^32,33^ in the active site mobile “flap”^34^. 2-Mercaptoethanol, which has previously been shown to inhibit urease from *Sporosarcina pasteurii*^34^, did not inhibit free JBU under the experimental conditions used here (Supplementary Table S2 and Table 2). However, 2-mercaptoethanol completely inhibited *K. pneumoniae* ureolysis at a concentration of 1 mM without affecting bacterial growth, indicating that this thiol specifically targets urease activity in the cell (Supplementary Table S1 and Supplementary Table S2). The related thiol cysteamine is known to inhibit urease activity, and at a concentration of 0.1 mM, it reduced the final pH of the JBU activity assay by 36.4 ± 1.1% compared to the uninhibited enzyme (Table 2)^35^. Cysteamine did not affect *K. pneumoniae* ureolysis except at high concentrations (Supplementary Table S1).

### Oxidation of the Mobile “Flap” Cysteine

2-Bromo-2-nitropropane-1,3-diol and potassium permanganate (KMnO_4_) are both strong oxidizers that can potentially oxidize the SH-group of the mobile “flap” cysteine of urease, rendering the enzyme inactive^36,37^. However, these types of compounds are able to oxidize many chemical groups, and they are therefore likely to be nonspecific. Both compounds were found to inhibit JBU at a concentration of 0.1 mM, and KMnO_4_ also had a good inhibitory effect on the ureolytic activity of *K. pneumoniae* (Supplementary Table S1 and Table 2). 2-Bromo-2-nitropropane-1,3-diol is marketed as an antimicrobial agent under the name *Bronopol*, and in this study, the antibacterial^37^ effect of the lowest concentration was strong enough to fully inhibit *K. pneumoniae* growth, making the anti-ureolytic activity of the compound less relevant (Supplementary Table S1).

### Michael Acceptors Covalently Modifying the Mobile “Flap” Cysteine

Several Michael acceptors are known to covalently bind to the sulfhydryl (-SH) group of the mobile “flap” cysteine^38^ of urease (Cysα319 in *K. pneumoniae* urease and Cys592 in JBU)^32,33^. These compounds inhibit ureolysis by locking the flap in a configuration that sterically hinders urea from reaching the catalytic Ni centre^39–41^. In the present study, quinones generally performed very well against the activity of JBU, but all showed quite low anti-ureolytic effects against *K. pneumoniae* (Table 2). The two quinones p-benzoquinone and 2,5-dimethyl-1,4-benzoquinone showed very high inhibition of free urease even at concentrations below 0.1 mM (Supplementary Table S2 and Table 2). The anti-ureolytic effect of 2,5-dimethyl-1,4-benzoquinone, *p*-chlorophenol, and N-(4-hydroxyphenyl)glycine on *K. pneumoniae* increased with higher concentrations of inhibitor, but the effect on *K. pneumoniae* never reached that on the pure enzyme (Supplementary Table S1 and Supplementary Table S2). Common to all the quinones tested here was complete inhibition of bacterial growth at the highest tested concentrations owing to the high reactivity and low selectivity of the inhibitors, which generally make them antibacterial compounds as well as good urease inhibitors against free urease (Supplementary Table S1 and Supplementary Table S2)^42^. However, in complex samples such as manure slurry or where toxicity is an issue, high reactivity and low selectivity are less beneficial.

**Figure 4:**
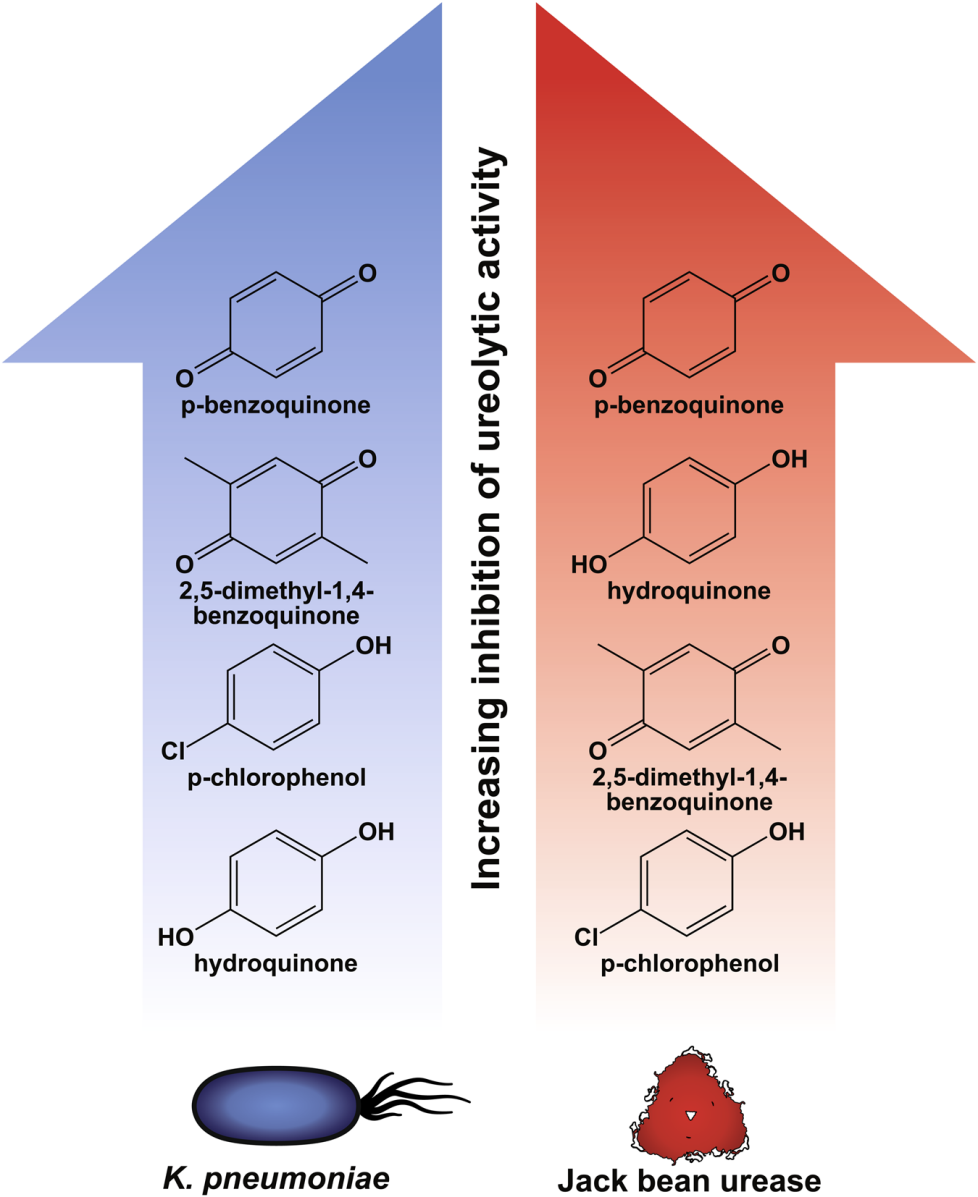
Structures of p-benzoquinone and p-benzoquinone derivatives inhibiting urease activity. The Michael acceptor p-benzoquinone and derivatives thereof have been ranked according to increasing inhibition of the ureolytic activity of *K. pneumoniae* (blue) and jack bean urease (red).

Among the tested quinones, p-benzoquinone was the most effective anti-ureolytic compound against both bacteria and free urease (Fig. 4). Of the tested p-benzoquinone derivatives, hydroquinone was notable for having no effect against *K. pneumoniae* ureolysis or growth at any concentration tested and for performing quite well against JBU, suggesting that it is either too reactive or cannot penetrate the cell membrane (Table 2 and Fig. 4). Hydroquinone and 2,5-dimethyl-1,4-benzoquinone are equally good inhibitors of JBU while p-chlorophenol is much less active against the pure enzyme. In contrast, 0.1 mM 2,5-dimethyl-1,4-benzoquinone and 0.1 mM p-chlorophenol are able to partly inhibit the ureolysis of *K. pneumoniae* with 2,5-dimethyl-1,4-benzoquinone being the better anti-ureolytic agent (Fig. 4, Supplementary Table S1 and Supplementary Table S2).

N-phenylmaleimide has not been previously described as an inhibitor of urease, but its ability to react with free thiols is well known^43^, and it induced 100% inhibition of JBU at a relatively low concentration (40 µM). Thus, N-phenylmaleimide performed better than the quinones tested here except for p-benzoquinone. At 0.4 mM, N-phenylmaleimide fully inhibited the growth of *K. pneumoniae*, indicating an antibacterial effect as well. Finally, N-(4-hydroxyphenyl)glycine inhibited JBU with a 45 ± 6.5% reduction in the final pH value at a very low concentration (1 µM) (Table 2), and at a 10-fold higher concentration, it was also capable of inhibiting *K. pneumoniae* ureolysis with an approximately 54% reduction in the final pH (Supplementary Table S1).

### Metal Ions Modifying the Mobile “Flap” Cysteine

Silver ions (Ag^+^) have been shown to be powerful inhibitors of urease^15^. A recently published crystal structure of *Sporosarcina pasteurii* urease inhibited by Ag^+^ indicated that two Ag^+^ ions are coordinated by the active site mobile “flap” Cys322 thiolate, a nearby thioether, and a histidine nitrogen^44^. As with the Michael acceptors, this modification also prevents the mobile “flap” from moving, thus inhibiting the activity of urease. In the present work, 0.1 mM Ag^+^ was added in the form of AgNO_3,_ and as expected, the catalytic activity of JBU was completely inhibited (Table 2). In the case of *K. pneumoniae*, this concentration of silver was found to be antibacterial (Supplementary Table S1). A potential issue with applying Ag^+^ as an inhibitor of urease activity in practical terms could be the formation of insoluble AgCl, which prevents the silver from reacting with enzymes, e.g., urease. However, the concentration of Cl^−^ in the M9U medium in the present study was 18.14 mM^20^, which did not prevent Ag^+^ from acting as an anti-ureolytic and antibacterial agent. However, in more complex solutions, it is unlikely that Ag^+^ will be present long enough to effectively inhibit urease, although it may kill bacteria.

### Nickel Chelators Affecting Nickel-uptake and Urease Activity

In the assay, two metal chelators were found to be able to sequester enough Ni^2+^ to inhibit the production of active urease by *K. pneumoniae*. The M9U medium contained 0.34 µM Ni^2+^ (as NiCl_2_), and the change in pH in the presence of 0.04 mM diethylenetriaminepentaacetic acid (DTPA) or 0.07 mM ethylenediaminetetraacetic acid (EDTA) was completely abolished relative to that in the uninhibited control (Table 2). Unexpectedly, the two chelators increased the ureolytic activity of purified JBU, which may be due to their binding of divalent metal ions other than Ni^2+^, such as Zn^2+^, which is known to reduce the activity of urease^16^. The compound 1,2,4-butanetricarboxylic acid (0.1 mM) reduced the rate of pH increase and the final pH change of JBU by 29.8 ± 11.8% and 25.4 ± 10.4%, respectively. Under the conditions applied here, 1,2,4-butanetricarboxylic acid completely inhibited the ureolytic activity of *K. pneumoniae* but only at a high concentration (10 mM), while only a small inhibition of ureolysis was observed at 1 mM (Supplementary Table S1). Interestingly, it has been proposed that 1,2,4-butanetricarboxylic acid is able to aid cellular uptake of Ni^2+^ by *E. coli* by acting as a metallophore for the extracytoplasmic solute-binding protein NikA of the NikABCDE canonical ABC importer^45^. Hu and Mobley (1993) suggested that the anti-ureolytic effect of L-cysteine on bacteria can be attributed to L-cysteine acting as a nickel chelator^46^. To further investigate the anti-ureolytic property of L-cysteine, this compound and two of its ester adducts (L-cysteine methyl ester and L-cysteine ethyl ester) were tested in ureolytic activity assays. At a concentration of 1 mM, L-cysteine, L-cysteine methyl ester, and L-cysteine ethyl ester completely inhibited the ureolytic activity of *K. pneumoniae* and increased the activity of JBU, similar to the results observed for EDTA and DTPA (Table 2). Thus, these results are consistent with previous observations showing that L-cysteine has an anti-ureolytic effect on bacteria.

To study the diverse effects of the different potential Ni^2+^-chelators on ureolysis, ten chelators were investigated further using a nickel uptake assay with the non-chelator urease inhibitor sodium fluoride as a control. The compounds were tested against two different nickel transporters, namely, the energy-coupling factor (ECF) transporter Nik(MN)QO from *R. capsulatus* and the NiCoT nickel transporter from *K. pneumoniae*, using *E. coli* XL1-Blue producing either of the two transporters recombinantly (Supplementary Fig. S1 for full results). Nik(MN)QO is a primary transporter requiring binding and hydrolysis of ATP for subunit rearrangements^47^, while NiCoT members may employ a secondary (uniport) mechanism. The compounds were thus screened against mechanistically different types of Ni transporters.

**Figure 5:**
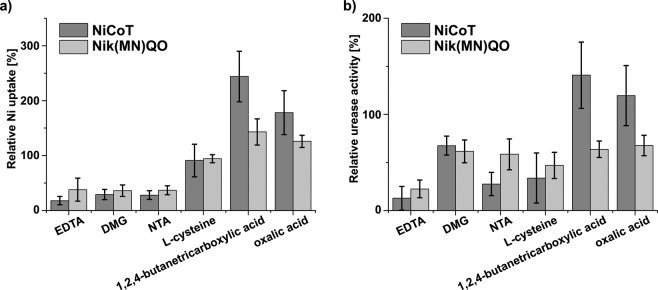
The effects of potential Ni^2+^-chelators on nickel uptake and urease activity relative to an uninhibited control. The effects of the six compounds EDTA, DMG, NTA, L-cysteine, 1,2,4-butanetricarboxylic acid and oxalic acid on Ni uptake (**a**) and urease activity (**b**) relative to an uninhibited control in assays using strain *E. coli* XL1-Blue harbouring a plasmid carrying either the gene encoding the Ni transporter NiCoT (from *K. pneumoniae*) or Nik(MN)QO (from *R. capsulatus*) and a plasmid with the urease operon (from *K. aerogenes*). (**a**) The effect of the six compounds on Ni uptake (pmol NiCl_2_/mg protein) shown as the mean percentage compared to the uninhibited control ±SD. (**b**) The effect of the six compounds on urease activity (mU/mg protein) shown as the mean percentage compared to the uninhibited control ±SD. *E. coli* expressing the NiCoT transporter (dark grey bars) or the Nik(MN)QO transporter (light grey bars) were used in both assays.

In the nickel uptake assay, five of the compounds either increased or decreased the uptake of Ni through both nickel transporters (Fig. 5a). The compounds EDTA, DMG, and NTA lowered the relative Ni uptake by the NiCoT transporter to 18 ± 8%, 29 ± 9% and 28 ± 8%, respectively (Fig. 5a). The same chelators reduced the relative Ni uptake by the Nik(MN)QO transporter to 38 ± 21%, 36 ± 11% and 37 ± 8%, respectively. With the exceptions of 1,2,4-butanetricarboxylic acid and oxalic acid, the remaining chelators did not significantly affect Ni uptake (Supplementary Fig. S1). Thus, 1,2,4-butanetricarboxylic acid and oxalic acid actually increased the relative Ni uptake through the NiCoT transporter to 244 ± 46% and 178 ± 40%, respectively (Fig. 5a). Ni uptake through Nik(MN)QO also increased in the presence of the two compounds but to a much lower degree (143 ± 24% and 126 ± 11% for 1,2,4-butanetricarboxylic acid and oxalic acid, respectively). Since no metallophore is involved in the binding of Ni^2+^ to the substrate-specific component NikM^48^, the two acids may increase the accessibility of Ni^2+^ in the LB medium used for the assay that contains a complex mixture of (metal-binding) compounds. Interestingly, the results from the nickel uptake assay were only partly in accordance with the results from the pH-based urease activity assay using *K. pneumoniae* (Supplementary Table S1 and Table 2).

In the assay using *K. pneumoniae*, EDTA strongly inhibited bacterial ureolysis, DMG had some effect, while NTA had no effect. In addition, oxalic acid reduced bacterial ureolysis (Supplementary Table S1 and Table 2) and inhibited purified JBU, unlike other chelators, such as EDTA or DTPA, indicating that it may have a different mode of action against urease aside from chelation (Supplementary Table S2). It was interesting that Ni uptake increased dramatically in the presence of 1,2,4-butanetricarboxylic acid (Fig. 5a), but this compound failed to increase ureolysis in the pH-based urease activity assay using *K. pneumoniae* (Table 2). Unexpectedly, L-cysteine had no effect on Ni uptake by either the NiCoT or Nik(MN)QO transporter but inhibited *K. pneumoniae* ureolysis by 100% (Fig. 5a and Table 2), indicating that the anti-ureolytic mechanism of L-cysteine and its ester adducts is more complicated than simple nickel chelation. Sissons and Yakub (2000) have shown that 1 mM L-cysteine suppresses the urease concentration in *Streptococcus salivarius*. They suggest that cysteine derived from protein hydrolysis may act as a signal indicating alkaline conditions and leading to a reduction in the expression of urease^6^.

To further investigate the actions of the five Ni^2+^ chelators as well as L-cysteine, nickel uptake measurements were performed with an indophenol-based urease activity assay. This urease activity assay was performed using the same *E. coli* XL1-Blue strain containing a plasmid with genes encoding either of the two nickel transporters (NiCoT or Nik(MN)QO) and an additional plasmid with the urease operon from *K. aerogenes* (Fig. 5b). The effect of EDTA on nickel uptake was reflected in urease activity. Thus, EDTA reduced the relative urease activity of NiCoT- and Nik(MN)QO-containing *E. coli* cells to 12.8 ± 12% and 22.5 ± 9% of the activity of the uninhibited control, respectively. The anti-ureolytic effect was not as pronounced for DMG, which reduced the relative urease activity to 67.5 ± 10% and 61.6 ± 12% of the activity of the control for NiCoT- and Nik(MN)QO-containing cells, respectively (Fig. 5b). The anti-ureolytic effect of NTA varied between the nickel transporters, as it reduced the relative urease activity to 27.6 ± 12% and 58.5 ± 16.1% of the activity of the control for NiCoT- and Nik(MN)QO-containing bacteria, respectively. These results correlate mostly with those from the pH-based urease activity assay, where the effect of EDTA was most prominent and DMG showed less anti-ureolytic activity. However, NTA showed no significant effect in the pH-based urease activity assay (Supplementary Table S1). The partially different results obtained with the two types of urease activity assays are likely due to the use of different bacteria (*K. pneumoniae* and the *E. coli* XL1-Blue strain) and the overall setup. According to the Ni uptake experiments, it appears that the increase in Ni uptake caused by 1,2,4-butanetricarboxylic acid and oxalic acid was more prominent for the NiCoT transporter than for the Nik(MN)QO transporter (Fig. 5a). The same trend can be seen from the indophenol-based urease activity assay, where the relative urease activity of the bacteria carrying the NiCoT transporter increased to 140.7 ± 34.5% and 119.5 ± 31.2%, while for the Nik(MN)QO-containing bacteria, the relative urease activity was reduced to 63.7 ± 8.6% and 67.6 ± 10.7% by 1,2,4-butanetricarboxylic acid and oxalic acid, respectively. In the indophenol-based urease activity assay, L-cysteine was found to reduce the urease activity of NiCoT- and Nik(MN)QO-containing cells to 33.8 ± 26.1% and 46.9 ± 13.6%, respectively (Fig. 5b). Together with the strong anti-ureolytic activity of L-cysteine observed in the pH-based urease activity assay (Table 2), these results support the hypothesis that L-cysteine reduces urease activity in a different manner than chelation of nickel.

### Protein precipitation and modulation of membrane permeability

At a concentration of 0.1 mM, the commercially available polyphenol tannic acid (TA) was found to reduce the final pH change caused by ureolytic *K. pneumoniae* by 18.4 ± 1.3% as well as slow the bacterial growth rate without affecting the final cell density (Supplementary Table S1 and Table 2). However, at a higher TA concentration (10 mM), no growth was detected (Supplementary Table S1). When 0.1 mM TA was applied to pure JBU, the inhibition was 100% relative to the uninhibited control. TA itself has not been shown to specifically inhibit urease, but polyphenols in general are known to precipitate proteins and other biomolecules, thus inhibiting a variety of enzymatic processes as well as altering the permeability of bacterial membranes^49–51^. The compound 1,2,3,4,6-penta-O-galloyl-β-D-glucopyranose is structurally related to TA and has been shown to inhibit *H. pylori* urease almost to the same level as the well-known urease inhibitor acetohydroxamic acid (AHA)^52^.

### Other Anti-Ureolytic Mechanisms

Gallic acid (GA), a monomer of TA, had moderate effect on the ureolysis of *K. pneumoniae* but reduced the final pH of the JBU activity assay by 64% at the relatively low concentration of 0.06 mM, suggesting that GA may not be able to easily cross the bacterial membrane but that GA interacts with pure urease. To the best of our knowledge, the function of GA as an inhibitor of urease has not been published previously. At high GA concentrations, a strongly coloured black complex was formed and was most likely iron(III)-gallate ([Fe^III^(GA)_3_)^53^. L-cysteine and its ester adducts showed no anti-ureolytic activity against pure urease but were all very efficient in inhibiting the urease activity of *K. pneumoniae* (Table 2 and Fig. 5b). These results, together with the observation that L-cysteine does not affect Ni uptake (Fig. 5a), indicate that the anti-ureolytic mechanism of L-cysteine and its ester adducts is not due to chelating effects, and they reduce the amount of active urease in the cells through an unknown mechanism. Sodium pyrrolidinedithiocarbamate was found to cause a non-ureolytic pH increase and showed bactericidal activity towards *K. pneumoniae*. The compound decreased the ureolytic activity of JBU, although the mechanism is currently not known (Table 2), but the anti-ureolytic activity of sodium pyrrolidinedithiocarbamate could be related to disulfide formation with the mobile “flap” cysteine residue or a reaction with the active site Ni atoms of urease.

### Different urease inhibitors for various applications

The results presented here illustrate the importance of choosing an anti-ureolytic compound that matches the intended purpose and application. For example, hydroxyurea is an excellent inhibitor of purified JBU but shows virtually no inhibition of *K. pneumoniae* ureolysis. Acetohydroxamic acid inhibits JBU and decreases the rate of *K. pneumoniae* ureolysis, but after 24 hours, the overall change in pH is relatively unaffected compared to the uninhibited control. Chelators such as EDTA do not inhibit free JBU but reduce *K. pneumoniae* ureolysis, consistent with the proposed Ni^2+^-chelation mechanism removing nickel needed for the assembly of active urease. Nickel chelators are most likely not efficient anti-ureolytic compounds in environments containing high levels of metal ions. For example, livestock manure, such as undiluted pig manure slurry, contains relatively high concentrations of metal ions, including approximately 10 µM Ni, leading to the need for quite high concentrations of chelators^54^.

**Figure 6:**
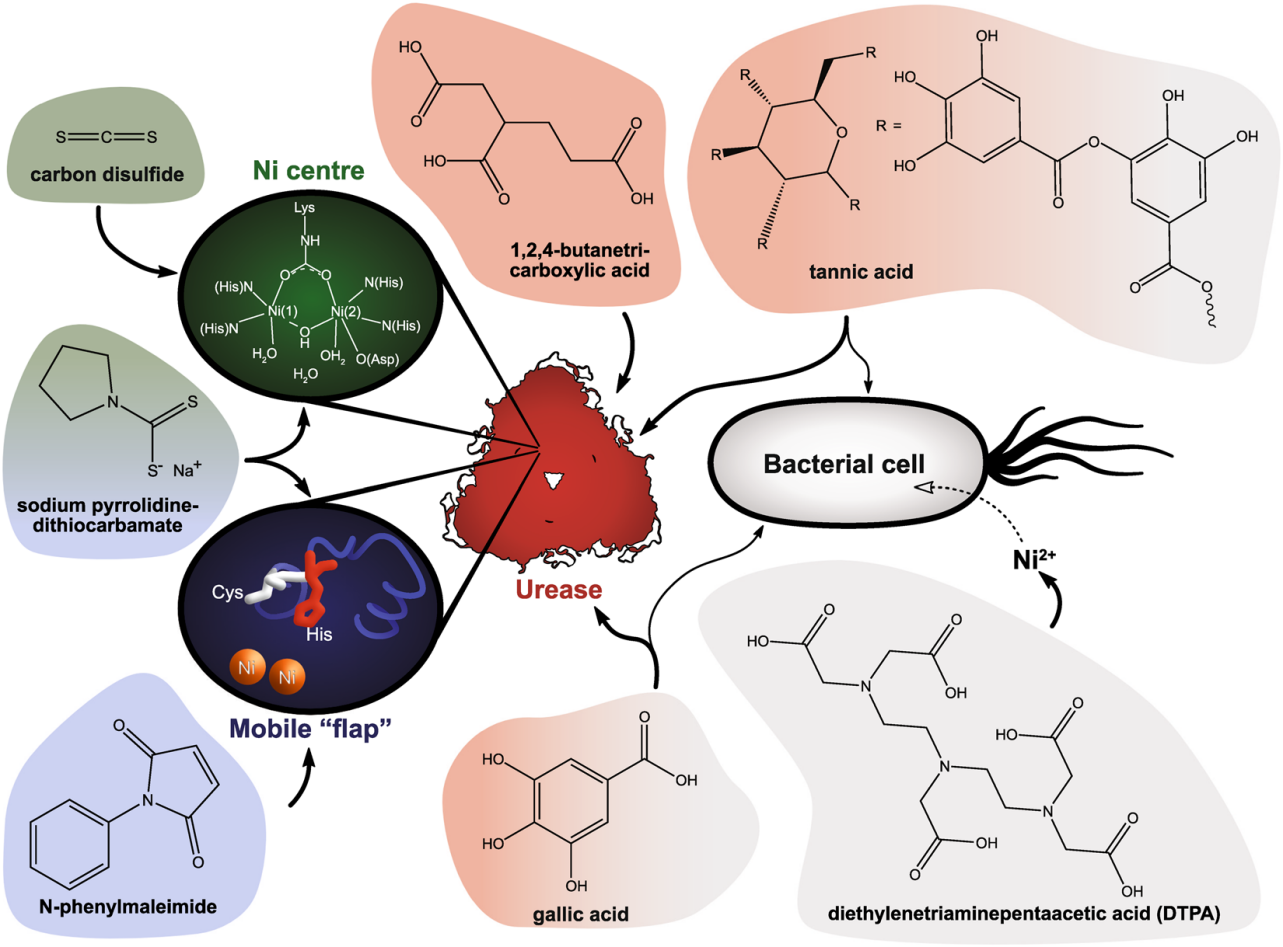
Structures of new anti-ureolytic compounds and their suspected targets of action. Seven compounds that have not previously been reported to have anti-ureolytic activity were identified in this study. Compounds are colour-coded based on their suspected targets of action. Compounds in grey are thought to affect the ureolytic activity of bacteria e.g. by sequestering Ni^2+^ or interacting with the membrane. Compounds in red appear to affect urease directly, but the mechanism is not known. Compounds in green and blue likely inhibit urease by binding to the Ni centre or the mobile “flap”, respectively. Compounds with several colours appear to affect urease/ureolysis in several ways. DTPA was found to have anti-ureolytic activity only against ureolytic bacteria (*K. pneumoniae*), while 1,2,4-butanetricarboxylic acid, carbon disulfide, N-phenylmaleimide and sodium pyrrolidinedithiocarbamate only showed anti-ureolytic activity against pure urease (JBU). Tannic acid and gallic acid predominantly had anti-ureolytic activity against cell-free urease but also reduced the ureolysis of bacteria. The illustration of the Ni centre was adapted from Krajewska 2009^2^.

With the present study, there is now for the first time a comprehensive comparison of multiple commercially available urease inhibitors and compounds with anti-ureolytic activity and their inhibitory effectivity on ureolysis from bacteria (*K. pneumoniae)* and purified urease (JBU) under identical conditions. Furthermore, seven compounds that have not previously been shown to have anti-ureolytic effects on either ureolytic bacteria or purified urease have been identified (Fig. 6).

DTPA is a strong metal chelator that can act as a multidentate ligand, potentially up to an octadentate ligand, or it can bind several metal ions but with lower denticity (Fig. 6). DTPA is related to EDTA, which is a hexadentate ligand, but due to the greater denticity, DTPA can be expected to show different stability constants and binding geometries compared to EDTA. In comparison, 1,2,4-butanetricarboxylic acid has a lower denticity than EDTA and lacks any nitrogen centres (Fig. 6). This makes chelation a less likely anti-ureolytic mechanism of 1,2,4-butanetricarboxylic acid, and this conclusion is supported by the fact that 1,2,4-butanetricarboxylic acid does not affect *K. pneumoniae* ureolysis, while it inhibits the ureolytic activity of JBU (Table 2). The observed significant inhibitory effect of tannic acid on JBU is most likely due to the ability of polyphenols to bind and precipitate proteins. However, it is less clear how tannic acid reduces the urease activity of *K. pneumoniae*, but it is probably a combination of altered membrane permeability and general protein binding and precipitation. Gallic acid, which can be viewed as a monomer of tannic acid, does not have a significant anti-ureolytic effect on *K. pneumoniae* but clearly inhibits the ureolytic activity of free urease at low concentrations (Table 2 and Fig. 6). The diverse anti-ureolytic properties of TA and GA are likely due to their different sizes (Fig. 6). N-phenylmaleimide most likely reacts with the mobile “flap” cysteine of urease, while sodium pyrrolidinedithiocarbamate may modify the mobile “flap” cysteine and/or directly bind to the active-site nickel atoms of urease in an unknown mechanism. Furthermore, both N-phenylmaleimide and sodium pyrrolidinedithiocarbamate were sufficiently antibacterial and fully inhibited the growth of *K. pneumoniae*. Finally, the small carbon disulfide molecule (Fig. 6) was able to inhibit JBU likely by binding nickel in the active site of urease. However, the inhibitory effect was only observed at the lowest concentration of inhibitor.

## Conclusion

Of the 71 commercially available compounds screened for anti-ureolytic activity against the ureolytic bacterium *K. pneumoniae* and purified jack bean urease, 30 compounds were found to reduce the final pH change and/or the rate of pH increase by more than 25% in a urease activity assay. Seven of these anti-ureolytic compounds have not previously been described to inhibit urease activity (Fig. 6). Additionally, selected chelators were characterized according to their ability to alter nickel uptake through the NiCoT and Nik(MN)QO nickel transporters as well as their effects on urease activity in a transformed *E. coli* strain. Interestingly, it appears that a decrease in nickel uptake does not necessarily translate to a similar reduction in urease activity as was observed in the case of EDTA versus DMG (Fig. 5). Furthermore, some compounds classed as chelators actually increased both nickel uptake and urease activity, as evidenced especially by 1,2,4-butanetricarboxylic acid. Finally, L-cysteine, which has previously been thought to reduce the urease activity of bacteria through nickel chelation, was found in the present study to decrease the urease activity of bacteria without affecting nickel uptake. This suggests that the inhibitory effect of L-cysteine and its ester adducts against bacterial ureolysis is not related to the chelation of nickel.

The results of the screening represent the first comprehensive comparison of different urease inhibitor classes using the same model systems, activity assays and experimental setup; therefore, we believe it should aid in the development of new anti-ureolytic compounds as well as the choice of which compounds to include in future studies of ureolytic organisms, urease and urease inhibitors. The development of novel inhibitors against ureolytic activity is important due to the widespread consequences of ureolysis associated with human health and environmental and economic challenges faced by society^12,55,56^.

## Materials and Methods

### Chemicals and equipment

Phenylphosphorodiamidate (PPDA), 97%, was purchased from Fisher Scientific (Roskilde, Denmark). N-(n-butyl)thiophosphoric triamide was purchased from Carbosynth (Compton, UK). Ethacrynic acid was purchased from Alfa Aesar (Karlsruhe, Germany). Ferric dimethyldithiocarbamate and 2,2´-thenoin were purchased from TCI Europe (Zwijndrecht, Belgium). All other chemicals were purchased from Sigma-Aldrich and used as received unless otherwise stated. All labware was purchased sterile or autoclaved before use. All solutions were autoclaved or filter-sterilized through sterile syringe filters with 20 µm pore sizes, and all work with sterile materials was carried out under laminar flow. Absorbance measurements were carried out with a Varioskan LUX plate reader (Thermo Scientific, USA) using flat bottom 96-well BRAND plates. Plates were sealed with optically clear AB-0812 Diamond Seal heat sealing tape using an ALPS30 heat sealer (Thermo Scientific, USA) prior to incubation in the plate reader. Nonlinear regression analysis was performed using OriginPro 9.0 software.

### Urease enzyme and bacteria

Purified jack bean (*Canavalia ensiformis*) urease was purchased from Sigma-Aldrich and dissolved in an aqueous 15 mM KH_2_PO_4_ solution at pH 6.8 to give a final concentration of 1.89 mg/ml, corresponding to 66.15 U/ml. The urease stock was stored at −20 °C. The final JBU concentration in the pH-based urease activity assay was 0.07 U/ml. The ureolytic bacterium *Klebsiella pneumoniae* subsp. *pneumoniae* (ATCC, 13882; DSM No., 30102) and the non-ureolytic bacterium *Escherichia coli* K12 MG1655 (ATCC, 700926; DSM No., 18039) were used in the pH-based urease activity assay. *Escherichia coli* XL1-Blue was used for the Ni uptake assay and the indophenol-based urease activity assay.

### *pH*-based urease activity assay

The bacterial and enzymatic urease activity assays were carried out in M9U medium as described previously^20^. Briefly, the assay consisted of a buffered 40 mM urea solution containing the pH indicator phenol red and the inhibitor to be tested. Stock solutions were prepared for each inhibitor at concentrations of either 100 mM or, in the case of low solubility, as concentrated as possible (Supplementary Table S1 and Supplementary Table S2). Each compound was screened at three concentrations (10×, 100× and 1000× dilutions of the stock) in triplicate against *K. pneumoniae* and JBU. Cultured *K. pneumoniae* cells or pure urease were added to the solution before plates were incubated in a plate reader. Overnight cultures of *K. pneumoniae* and *E. coli* were prepared in M9U medium. After approximately 12 hours of growth the cells from overnight cultures were pelleted by centrifugation, the supernatant removed and new M9U added to yield an OD600 of 0.125. The bacterial suspension was added to each well (80 µl) along with 100 µl M9U and 20 µl inhibitor solution (or water in the controls) to yield a final volume of 200 µl. The final OD600 in each well was 0.05^20^. The absorbance at 557 nm and 630 nm (A557 and OD630) was measured every 15 minutes for 24 hours. Inhibitor and bacteria/urease were not preincubated prior to the addition of urea or at the beginning of the measurements.

The increase in optical density at 630 nm (OD630) reflects bacterial growth, while the difference between the absorbance at 557 nm and 630 nm relates to pH (A557 – A630). In the case of ureolytic bacteria or urease in urea solution, the increase in pH can be ascribed to the production of alkaline NH_3_. In the bacterial assay, inhibitors were evaluated based on three parameters: onset of pH increase, final pH change, and rate of pH increase. The onset of the pH increase was defined as the point in time where A557 (deprotonated phenol red) began to increase. The final pH change was determined as the final A557 – A630 value. The rate of pH increase was defined as the slope of the pH increase using a version of the Gompertz fit as previously described^20^. The Gompertz equation (Eq. (1)) is used to describe data resembling a typical microbial growth curve with lag, exponential, and stationary phases.

1 $$y=a{e}^{-{e}^{(-k(x-{x}_{c}))}}$$

In Eq. (1), y is the expected absorbance as a function of time, x is time, x_c_ represents time at inflection (parameter related to length of lag-phase), *a* is the absorbance at the stationary phase, and *k* is the growth rate coefficient^57^. The influence of each inhibitor on bacterial growth was also evaluated by identifying the length of the lag phase (onset of exponential growth), the growth rate in the exponential phase, and the final OD630 (Supplementary Table S1).

For the enzymatic urease activity assays, the inhibitors were evaluated based on two parameters: the initial rate of pH increase found by linear regression of the increase in A557 during the first 90 min of incubation and the final pH change defined as the final A557 value. The onset of pH increase was not found to be a useful parameter in the enzymatic assays, as the increase in A557 was initiated within the first two measurements (<15 min) for most of the inhibitors.

### Nickel uptake assay

The nickel uptake assay was carried out as described previously^58–60^. Briefly, strain *E. coli* XL1-Blue containing either plasmid pRcNik(MN)QO_F_ encoding Nik(MN)QO from *R. capsulatus*^58^ or plasmid pCH675-KP containing the NiCoT gene from *K. pneumoniae*^61^ was grown overnight in lysogeny broth (LB) supplemented with 100 µg/ml ampicillin. Both plasmids, pRcNik(MN)QO_F_ and pCH675-KP, contained ampicillin resistance genes. After a 1:100 dilution of the overnight cultures in fresh LB containing 100 µg/ml ampicillin, 1 mM IPTG, 60 nM radiolabelled^62^ NiCl_2_ and either no inhibitor, 1 mM 1,2,4-butanetricarboxylic acid, 0.1 mM 1,4,8,11-tetraazacyclotetradecane (cyclam), 1 mM L-cysteine, 0.5 mM dimethylglyoxime (DMG), 0.07 mM EDTA, 0.1 mM imidazole, 0.05 iminodiacetic acid, 0.05 mM N-(2-acetamido)iminodiacetic acid (ADA), 0.3 mM nitrilotriacetic acid (NTA), 1 mM oxalic acid or 0.5 mM NaF were added. The cell culture was incubated at 37 °C with shaking for 7 hours, after which 2 ml of cell culture was harvested by centrifugation (4 min, 8000 × *g*) and washed twice with 50 mM Tris-hydrochloride (pH 7.5). Subsequently, the cells were resuspended in 200 µl of 50 mM Tris-hydrochloride (pH 7.5), and the OD_578_ was measured. Nickel accumulated in the cells was quantified using a Tri-Carb 4910 TR liquid scintillation counter (Perkin Elmer). All experiments were performed in triplicate.

### Indophenol-based urease activity assay

The assay was based on a previously described method^60,63^ that has been modified to use a microtiter plate setup. Briefly, the *E. coli* XL1-Blue strain containing a modified version of plasmid pKAU17 carrying the urease operon from *Klebsiella aerogenes*^62^ but conferring streptomycin resistance instead of the original ampicillin resistance was co-transformed with either pRcNik(MN)QO_F_ or pCH675-KP encoding the nickel transporters Nik(MN)QO and NiCoT, respectively. Both nickel transporter-encoding plasmids carried ampicillin resistance genes. The double-transformed cells were grown overnight in LB in the presence of 100 µg/ml ampicillin and 50 µg/ml streptomycin. The overnight cultures were diluted 1:100 in fresh LB containing 100 µg/ml ampicillin, 1 mM IPTG, 500 nM NiCl_2_, and either of the inhibitors mentioned above in the description of the nickel uptake assay. Subsequently, the cells were grown for 10 hours at 37 °C with shaking before 2 ml of cells was harvested by centrifugation (5 min, 8000 × *g*) and washed twice with 35 mM sodium/potassium phosphate buffer (pH 7). The cells were then suspended in the same phosphate buffer with 0.15 mM cetyltrimethylammonium bromide (CTAB) to an OD_578_ of approximately 3, and 0.5 ml or 0.05 ml of the cell suspension was diluted in 0.15 mM CTAB to reach a total volume of 2 ml. The resulting solutions of lysed cells were incubated for 5 min at 37 °C, and urea hydrolysis was initiated by the addition of urea to a final concentration of 5 mM. Samples of 16 µl were withdrawn from the reaction after 5, 10, 20, and 30 min and transferred to a microtiter plate. The ammonia concentration in each plate well was determined using the indophenol-hypochlorite reaction^64^. The final volume of the indophenol reaction mixture was 250 µl, which consisted of 0.25 M sodium salicylate, 0.1 mM sodium nitroprusside, 68 mM NaOH, 60 mM sodium citrate, and 11 mM sodium hypochlorite. The amount of formed ammonia was determined by measuring the absorbance at 650 nm after 1 hour at 37 °C. All experiments were performed in triplicate.

## Supplementary information

Supplementary table S1

Supplementary table S2

Supplementary figure S1
