# Supplementary material for: Inhibition of urease activity by different compounds provides insight into the modulation and association of bacterial nickel import and ureolysis
Source: Sci Rep. 2020 May 22;10:8503. doi: 10.1038/s41598-020-65107-9 (PMC7244745; doi:10.1038/s41598-020-65107-9)
Supplement: Supplementary file 3 — Supplementary figure S1 [file 41598_2020_65107_MOESM3_ESM.docx]

**Inhibition of urease activity by different compounds provides insight into the modulation and association of bacterial nickel import and ureolysis**

### Simon Svane^‡^, Jens Jakob Sigurdarson^‡^, Friedrich Finkenwirth^†^, Thomas Eitinger^†^ and Henrik Karring^‡^*

### ‡Department of Chemical Engineering, Biotechnology and Environmental Technology, University of Southern Denmark, Campusvej 55, 5230 Odense M, Denmark. * hka@kbm.sdu.dk

### †Institut für Biologie/Mikrobiologie, Humboldt-Universität zu Berlin, Unter den Linden 6, 10099 Berlin, Germany.


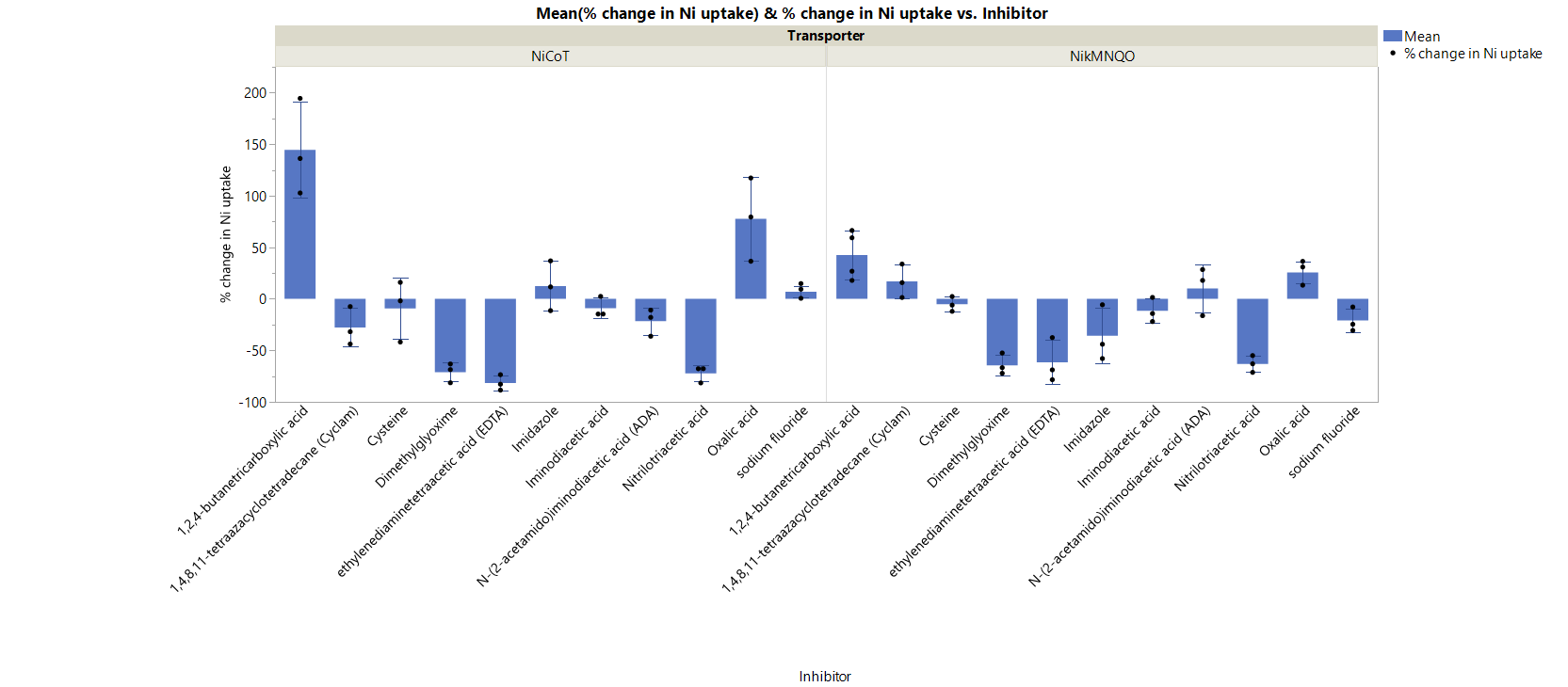


**Supplementary Figure S1. The effects of ten potential Ni^2+^-chelators and sodium fluoride in a nickel uptake assay against nickel transporters NiCoT and Nik(MN)QO**. The effect of the ten potential Ni chelators 1,2,4-butanetricarboxylic acid, 1,4,8,11-tetraazacyclotetradecane (Cyclam), L-cysteine, dimethylglyoxime, ethylenediaminetetraacetic acid (EDTA), imidazole, iminodiacetic acid, N-(2-acetamido)iminodiacetic acid (ADA), nitrilotriacetic acid, and oxalic acid, as well as the non-chelator urease inhibitor sodium fluoride, on Ni uptake by *E. coli* XL1-Blue recombinantly producing the urease of *K. aerogenes* and NiCoT of *K. pneumoniae* (left panel) or Nik(MN)QO of *R. capsulatus* (right panel). The effect on Ni uptake (pmol NiCl_2_/mg protein) is shown as the mean (blue bars) ±SD relative to the uninhibited control.
